# The moderating effect of psychosocial factors in the relation between neighborhood walkability and children’s physical activity

**DOI:** 10.1186/s12966-016-0452-0

**Published:** 2016-12-09

**Authors:** Sara D’Haese, Freja Gheysen, Ilse De Bourdeaudhuij, Benedicte Deforche, Delfien Van Dyck, Greet Cardon

**Affiliations:** 1 Faculty of Medicine and Health Sciences, Department of Movement and Sports Sciences, Ghent University, Watersportlaan 2, 9000 Ghent, Belgium; 2 Research Foundation Flanders (FWO), Egmontstraat 5, Brussels, 1000 Belgium; 3 Department of Public Health, Ghent University, De Pintelaan 185, 9000 Ghent, Belgium; 4 Department of Human Biometrics and Biomechanics, Vrije Universiteit Brussel, Brussels, Belgium

**Keywords:** Child, Neighborhood, Psychosocial, Interactions, Physical activity

## Abstract

**Background:**

The study aimed to investigate if psychosocial factors moderate the association between objective walkability and different domains of children’s physical activity (PA). A second aim of the study was to investigate the direct associations between psychosocial factors and children’s PA. Based on previous literature, it was hypothesized that walkability would be more strongly related to PA among children with negative psychosocial profiles.

**Methods:**

Data were collected between December 2011 and May 2013 as part of the Belgian Environmental Physical Activity Study in children (BEPAS-child). In total, data from 494 children and one of their parents were included in the study. Children wore an accelerometer for 7 consecutive days and together with one of their parents, they completed the Flemish Physical Activity Questionnaire. Parents filled out a questionnaire concerning their child’s psychosocial factors toward PA (i.e. parental attitude toward their child’s PA, parental social norm toward their child’s PA, parental support, friend support, children’s self-efficacy, and perceived benefits and barriers toward sports and PA). Neighborhood walkability was calculated using geographical information systems (GIS). Multilevel cross-classified analyses were conducted.

**Results:**

Of the 42 investigated interactions between neighborhood walkability and psychosocial factors in relation to PA among children, only 7 significant interactions were found of which 3 were only significant among children from low-income neighborhoods.

Parental support and self-efficacy were positive correlates of children’s PA in high- and low-income neighborhoods independent of the level of walkability, but effect sizes were small.

**Conclusions:**

The hypothesis that walkability would be more strongly related to PA among children with negative psychosocial profiles could not be confirmed and in general, psychosocial factors and objective walkability did not interact in relation to children’s PA. Focusing on parental support and self-efficacy towards PA can possibly cause small effects on children’s PA in both high- and low-walkable neighborhoods, as well as in high- and low-income neighborhoods.

**Electronic supplementary material:**

The online version of this article (doi:10.1186/s12966-016-0452-0) contains supplementary material, which is available to authorized users.

## Background

Despite the numerous health benefits of being sufficiently physically active during childhood [1], many children do not meet the PA guidelines of engaging daily in 60 min of moderate- to vigorous-intensity physical activity (MVPA) [2]. Therefore, it is necessary to obtain insight into physical activity (=PA) determinants among children. When the PA determinants are identified, focusing on these factors in future interventions may result in increased PA levels among children [3]. Ecological models state that PA can be explained by individual (e.g. psychological factors) as well as environmental (e.g. neighborhood characteristics, social environment) factors [4]. To date, the direct association between psychosocial factors (e.g. parental support), the neighborhood environment (e.g. walkability) and children’s PA has been thoroughly investigated [5–11].

In review studies, self-efficacy, parental PA (for boys), and parental support [10] were positively associated and perceived barriers toward PA [11] were negatively associated with children’s PA. On the other hand, the association between objective neighborhood walkability (characterized by residential density, street connectivity and land use mix diversity [12]) and children’s PA is less univocal [5, 13–17]. In a Belgian study with the current 9- to 12-year-old study sample, objective walkability was positively related to walking for transportation during leisure and was negatively related to sports during leisure only in low-income neighborhoods [17]. Further, no direct associations were found between objective walkability and active transportation to school (=ATS), cycling for transportation during leisure and objective MVPA on weekend- and weekdays [17]. Thus, in contrast to adult studies, in which higher walkability has been consistently related to more PA [18–21], only few direct associations between objective walkability and children’s PA were found and these associations were dependent on the domain of PA.

According to ecological models, it is likely that factors at different levels (e.g. walkability at the environmental level and psychological factors at the individual level) of the ecological model for PA interact with each other [4]. For example, it is possible that high walkability is related to more cycling for transportation during leisure, only among children with negative psychosocial profiles toward PA and that cycling levels among children with positive psychosocial factors are high, irrespective of their neighborhood walkability. However, the ecological model for PA does not specify which interactions between which factors can be expected or which interactions are most important to explain children’s PA [4]. Therefore, it is important to investigate which interactions exist between objective walkability and several psychosocial factors in relation to different domains of children’s PA. Several interactions between psychosocial factors and walkability in relation to PA were already identified among adolescents [22], adults [23, 24] and older adults [25, 26]. Among Belgian adolescents, it was found that in low-income neighborhoods, neighborhood walkability was positively associated with PA among adolescents who perceived many barriers and few benefits, while for adolescents who perceived few barriers and many benefits, the PA level was high, irrespective of neighborhood walkability [22]. Among Belgian adults, it was found that living in a high walkable neighborhood was associated with taking more steps, especially among adults with a preference for passive transport and/or a low intention to walk or cycle [24]. Among Belgian older adults walkability was positively associated with recreational walking in those with high self-efficacy [26]. However, to our knowledge, these interactions were not investigated among children yet. More insight into how neighborhood walkability and different psychosocial characteristics interact in relation to children’s PA, can help to identify groups in need of targeted interventions and to develop effective interventions to increase children’s PA.

Therefore, the aim of this study was to investigate if psychosocial factors moderate the association between walkability and different domains of children’s PA. Based on the results of previous studies among adolescents and adults, it was hypothesized that walkability would be more strongly related to children’s PA when children have a negative psychosocial profile (e.g. children who perceive many barriers and few benefits) toward PA, whereas children with more positive psychosocial factors engage in high levels of PA, irrespective of their neighborhood walkability. More specifically, based on previous research among children [17], positive associations are expected between walkability and active transportation to school, walking and cycling for transportation during leisure and MVPA on week- and weekend days among children with negative psychosocial profiles. Negative associations are expected between walkability and sports during leisure, among children with negative psychosocial profiles. Among children with more positive psychosocial profiles, it is expected that walkability is less important to explain their PA. As in previous analyses, walkability was only associated to PA among children living in low-income neighborhoods, analyses were stratified for low- and high-income neighborhoods when a significant three-way interaction between walkability, neighborhood income and psychosocial factors was found in relation to PA.

A second aim of this study was to describe the main effects of psychosocial factors in relation to different domains of children’s PA. It was hypothesized that having a more positive psychosocial profile toward PA would be related to more PA among children.

## Methods

### Procedure

**Fig. 1:**
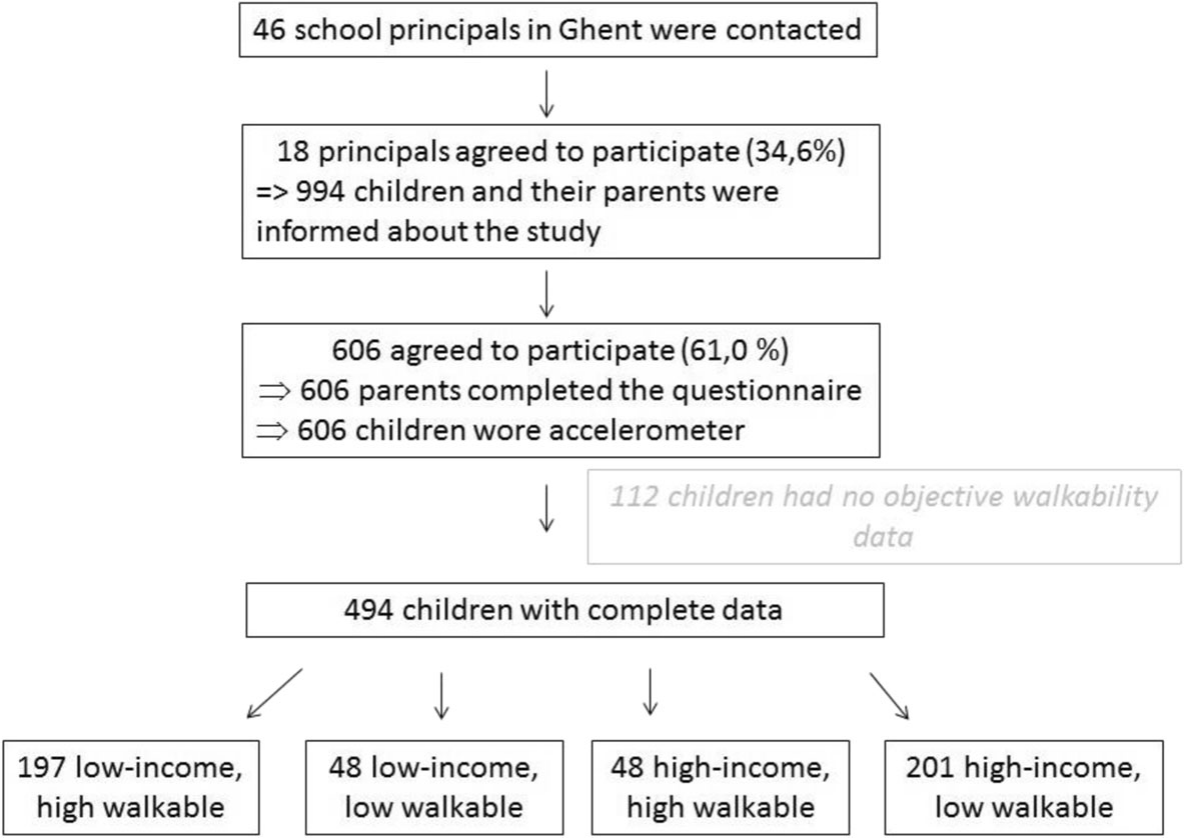
Flowchart of the data-collection

Data were collected between December 2011 and May 2013 as part of the Belgian Environmental Physical Activity Study in children (BEPAS-child). Principals (*n* = 46) from primary schools in Ghent (237000 inhabitants, 15685 km^2^) were asked to participate. In total, 18 (34.6%) agreed and gave written informed consent. All children and their parents from fourth, fifth and sixth grade (*n* = 994) were informed about the study and 606 parents (61.0%) gave written informed consent. Due to practical limitations, objective walkability data were only collected for children living in Ghent. Therefore, of these 606 children who participated, 112 children were excluded as no objective walkability data were available (69 children did not live in Ghent and 43 parents did not fill out children’s home address in the questionnaire). This resulted in a final sample of 494, 9- to 12-year-old children (Fig. 1).

Children were asked to wear an accelerometer for 7 consecutive days, to fill out a questionnaire at school and one of the parents was asked to fill out a questionnaire together with his/her child. The Ethics Committee of the Ghent University Hospital approved the study.

### Measurements

#### Demographic variables

Sex was derived from children’s questionnaire and children’s age from the parental questionnaire. Educational attainment of both parents was used as a proxy for family socio-economic status (SES). Parents were asked to report their level of education (response options: primary school education, vocational, technical, general or art secondary education, college education or university education). Families were classified as high SES families if the educational level of at least one parent was of a college or university education level; otherwise they were classified as low/medium SES families. Educational attainment was used as a proxy for family SES, as educational attainment is easy to measure and is fairly stable in early adulthood, and higher levels of education are usually associated with better jobs, housing, neighborhoods, working conditions and higher incomes [27] which are usually related to higher SES.

#### Physical activity

PA was measured using two complementary methods: accelerometry and questionnaires. These methods are not interchangeably as they are not assessing the same thing [28]. Accelerometry was used to determine children’s overall PA in an objective way by measuring accelerations of the body. The Flemish Physical Activity Questionnaire was used to determine PA among children in different contexts.

**Objective MVPA** was determined by accelerometers. Children wore an Actigraph^TM^ GT1M, GT3X or GT3X+ accelerometer (15 s epoch) during waking hours for 7 consecutive days. Strong agreement was found between these activity monitors for measuring children’s MVPA [29], making it acceptable to use different models within a given study. The accelerometer was worn on the right hip. Accelerometer data were screened, cleaned and scored using data-reduction software MeterPlus 4.2. Periods of 20 min of consecutive zeros or more were removed and defined as non-wear time [30, 31]. Non-wear time activity diaries were provided to register activities for which the accelerometer was removed and were used to replace the consecutive number of zeros by the corrected minutes MVPA [32]. MVPA was calculated using the cutpoints of Evenson (>2296 counts per minute was defined as MVPA), as these cutpoints were recommended in a comparative validity study [33, 34]. Children were included in the study if they had at least 2 weekdays with minimum 10 h wear time or 1 weekend day with minimum 8 h wear time [35].

**Children’s reported PA** was assessed with the Flemish Physical Activity Questionnaire (FPAQ). Parents were asked to fill out the questionnaire at home together with their child and to report their child’s PA levels in a usual week. This questionnaire has been shown to be a reliable and reasonably valid instrument to assess different dimensions of PA in children, especially when completed with parental assistance [36]. The number of minutes per day of walking and cycling for transport during leisure, ATS and sports during leisure were derived from the questionnaire.

#### Neighborhood variables

##### Neighborhood income

Ghent consists of 201 statistical sectors (the smallest administrative entities for which statistical data are available). Median annual household income data (National Institute of Statistics–Belgium, 2008) were used to determine neighborhood income of the different statistical sectors. Neighborhoods were characterized as low-income (income <€22,359) or high-income (income ≥€22,359) neighborhoods based on the median.

##### Walkability

Objective neighborhood walkability was calculated using a geographical information system (GIS) database. Geographical data were obtained from the Service for Environmental Planning in Ghent in 2012.

Residential density, intersection density and land use mix diversity of each neighborhood (i.e. statistical sector) were determined and z-scores were calculated. Walkability was calculated as follows: walkability = (2*z-connectivity) + (z-residential density) + (z-land use mix). Because no data of ‘retail floor area’ were available, this was omitted from the original formula of Frank and colleagues [37]. Residential density was calculated using the ratio of residential units to the land area devoted to residential use. Connectivity was represented by the ratio of the number of intersections (3 or more streets) to the land area. Land use mix indicated the degree of diversity of land use types. Five land use types were considered: residential, retail, office, institutional, and recreational. Neighborhoods were characterized as low walkable or high walkable, based on the median.

#### Psychosocial factors

Parental attitude toward their child’s PA, parental social norm toward their child’s PA, parental support, friend support, and self-efficacy, perceived benefits and barriers toward sports and PA were parental reported, as parents are often seen as the main decision makers for their child. Questions were answered from parents’ viewpoint, concerning children’s psychosocial factors. Questions to assess psychosocial factors were derived from previous studies among adults and adolescents [22, 38–42]. These psychosocial factors were derived from the ASE-model by De Vries et al. [43]. The predictive validity and reliability of these items has been demonstrated previously among adolescents and adults [44, 45]. Table 1 gives an overview of the content and response options of the psychosocial factors. The scores (range 1–5) on the different items per factor were summed, with a higher score representing better psychosocial factors toward PA. Cronbach Alpha’s ranged from 0.71 until 0.85. No reliability data were available for these factors measuring children’s psychosocial characteristics reported by their parents.

**Table 1:** Content and response options of the psychosocial factors

	Content of the items	Response options
Parental attitude toward their child’s physical activity (1 item)	I think that being physically active and doings sports for my child is:	very unimportant (=1), unimportant (=2), sometimes important/sometimes unimportant (=3), important (=4), very important (=5)
Parental support (6 items: Cronbach Alpha = 0.772)	How frequently … -…do you encourage your child to be active and do sports? -…are you physically active or doing sports together with your child? -…do you bring your child to the place where he/she sports, are you going to watch or support? -…are you watching or cheering your child while he/she sports? -…do you offer your child to be physically active together? -…do you say your child is doing well?	Never (=1), seldom (=2), sometimes (=3), often (=4), very often (=5)
Friend support (1 item)	How frequently are your child’s friends or siblings physically active together with your child?	Never (=1), seldom (=2), sometimes (=3), often (=4), very often (=5)
Parental social norm toward their child’s physical activity (1 item)	I think my child has to engage regularly in physical activity.	strongly disagree (=1), somewhat disagree (=2), neither agree or disagree (=3), somewhat agree (=4), strongly agree (=5)
Self-efficacy (4 items: Cronbach Alpha = 0.849)	I am sure my child will be physically active even if… -… he/she has to get up early. -… his/her friends want to do something else. -… he/she has a lot of work for school -… it is exhausting and difficult.	
Benefits (6 items: Cronbach Alpha = 0.713)	My child believes that being physically active and doing sports is important because… -… his/her condition and health will improve. -… he/she get in contact with (new) friends -… he/she enjoys being physically active -… he/she can show that he/she is better than others -… he/she does not get bored if he/she is physically active -… he/she lose weight and his/her body becomes more beautiful	
Barriers (8 items: Cronbach Alpha = 0.801)	My child cannot be engaged in sports… -… due to lack of time. -… because he/she does not enjoy sports. -… because he/she is not good in doing sports. -… because he/she does not always have transport to activities. -… because he/she is not allowed by his/her parents. -… because there are no sport facilities in our neighborhood. -… because it is too expensive. -… because there is nobody (no friends or family) who wants to accompany my child.	

### Analyses

Descriptive characteristics of the sample were analyzed using SPSS20. PA variables were logarithmically transformed to improve normality. After the transformation, skewness values were lower than |0.7| for al PA variables, except for walking for transportation during leisure (skewness −0.844). Linear regression analyses were conducted in MLwiN2.32. Multilevel modeling was used to take into account clustering of children within classes within schools; and schools, classes and neighborhoods were treated as cross-classified. Model parameter estimates were obtained via Markov Chain Monte Carlo procedures applying an orthogonal parameterization [46]. Before multilevel regression analyses were conducted, multicollinearity within psychosocial factors was checked by conducting Pearson’s correlations in SPSS20. The magnitude of the correlation coefficients did not exceed 0.60, indicating that multicollinearity was not present. Given that the association between neighborhood walkability and children’s may differ between low- versus high-income neighborhoods [17], preliminary analyses, examining three-way interactions between neighborhood walkability, neighborhood income and psychosocial factors were conducted for the different outcome measures. Significant three-way interactions were found in relation to active transportation to school and MVPA on weekend days. Therefore, the sample was stratified according to high- and low-income neighborhoods when these outcome measurements were investigated.

All psychosocial variables were centered around their mean and analyses were conducted in two consecutive steps. In a first step, for each PA measure, moderating (cross-product term of walkability and each psychosocial factor) and main effects were calculated separately for each psychosocial variable and walkability. In a second step, a multivariable model was built, including all main and interaction terms yielding *p* < 0.10 in the first step. All analyses were controlled for accelerometer wear time (if relevant), family SES, sex and age of the child.

Because regression coefficients represented relationships with logarithmically transformed PA variables, predicted weekly minutes of PA were calculated from MLwiN’s customized prediction window [47]. The predicted values were calculated with all covariates fixed at their mean. To visualize moderating effects, the predicted PA measure was plotted against the mean -1 standard deviation and the mean +1 standard deviation of the corresponding psychosocial variable at low and high walkability.

Local effect sizes of the interaction terms and significant terms were determined by calculating Cohen’s f^2^ effect sizes, which is an effect size measure to use in the context of multiple regression [48]. Cohen’s f^2^ effect sizes lower than 0.02 were considered very small, values between 0.02 and 0.15 are considered small, effect sizes between 0.15 and 0.35 are considered moderate and effect sizes larger than 0.35 are considered large [49].

*P* < 0.05 was considered as significant with exception for the interaction terms were it was set at *p* < 0.10 [50].

## Results

### Descriptive characteristics

In total, 45.1% of the children were boys and 37.1% had low family SES and the mean age was 10.9 ± 0.9 years. Parental reported psychosocial characteristics toward PA were generally high (Table 2).

**Table 2:** Descriptive characteristics of the sample

	Overall	Low-income neighborhoods		High-income neighborhoods	
		High walkable	Low walkable	High walkable	Low walkable
*N*	494	197	48	48	201
Sex (% boys)	45.1	46.7	50.0	45.8	42.3
Age (years)	10.93 ± 0.90	11.02 ± 0.93	10.96 ± 0.95	11.01 ± 0.90	10.81 ± 0.86
Family SES (% low SES)	37.1	51.1	42.6	20.8	26.4
Psychosocial factors (mean ± SD)					
Parental attitude (/5)	4.46 ± 0.64	4.36 ± 0.70	4.54 ± 0.58	4.50 ± 0.55	4.52 ± 0.61
Parental support (/5)	3.54 ± 0.74	3.39 ± 0.84	3.59 ± 0.67	3.71 ± 0.65	3.64 ± 0.66
Friend support (/5)	3.54 ± 1.02	3.40 ± 1.04	3.60 ± 1.12	3.41 ± 1.07	3.69 ± 0.94
Parental social norm (/5)	4.48 ± 0.76	4.29 ± 0.92	4.63 ± 0.53	4.59 ± 0.62	4.59 ± 0.63
Self-efficacy (/5)	3.39 ± 0.93	3.31 ± 0.95	3.39 ± 0.87	3.45 ± 0.93	3.45 ± 0.93
Benefits (/5)	3.48 ± 0.68	3.56 ± 0.74	3.47 ± 0.64	3.34 ± 0.73	3.44 ± 0.62
Barriers (/5)	1.83 ± 0.67	2.01 ± 0.77	1.83 ± 0.60	1.70 ± 0.51	1.71 ± 0.60
Physical activity [17] (mean ± SD, mins/day)					
Active transportation to school (*n* = 483)	5.1 ± 7.7	5.7 ± 8.6	3.7 ± 6.3	4.8 ± 8.8	4.8 ± 7.2
Walking for transportation during leisure (*n* = 484)	6.6 ± 11.6	11.3 ± 14.0	3.5 ± 7.9	4.6 ± 9.3	3.3 ± 8.1
Cycling for transportation during leisure (*n* = 485)	4.7 ± 9.1	5.0 ± 9.8	3.9 ± 5.5	5.3 ± 10.2	4.6 ± 8.9
Sports during leisure (*n* = 485)	20.2 ± 20.2	16.2 ± 19.1	25.4 ± 24.4	22.1 ± 18.2	22.4 ± 20.1
MVPA weekday (*n* = 409)	60.2 ± 23.5	56.0 ± 23.2	63.6 ± 19.7	64.2 ± 24.2	60.3 ± 24.3
MVPA weekend day (*n* = 389)	50.0 ± 30.6	47.1 ± 26.8	41.5 ± 24.1	54.8 ± 32.8	53.6 ± 33.9

### Interactions between psychosocial factors and objective walkability in relation to PA

An overview of the bivariate interaction- and main effects of psychosocial factors and walkability in relation to PA is given Table 3. The multivariate associations are presented in Table 4 and are described below.

**Table 3:** Bivariate associations between psychosocial factors, walkability and children’s PA

	Active transportation to school	Walking for transportation during leisure	Cycling for transportation during leisure	Sports during leisure	MVPA weekday	MVPA weekend day
	B(SE) 95% CI *p*	B(SE) 95% CI *p*	B(SE) 95% CI *p*	B(SE) 95% CI *p*	B(SE) 95% CI *p*	B(SE) 95% CI *p*
Psychosocial x walkability (ref = low)						
attitude x walkability	HI: 0.067 (0.155) −0.24; 0.37 0.667 LI: −0.355 (0.150)* −0.65; −0.06 0.018	0.124 (0.072) ^¥^ −0.02; 0.27 0.085	−0.017 (0.074) −0.16; 0.13 0.816	−0.156 (0.085) ^¥^ −0.32; 0.01 0.068	−0.037 (0.024)* −0.08; −0.01 0.130	−0.053 (0.049) −0.13; 0.06 0.282
Parental support x walkability	−0.054 (0.068) −0.19; 0.08 0.433	0.018 (0.064) −0.11; 0.14 0.780	0.043 (0.063) −0.08; 0.17 0.501	−0.158 (0.070)* −0.30; −0.02 0.025	−0.036 (0.021) ^¥^ −0.08; 0.01 0.080	−0.031 (0.042) −0.11; 0.05 0.455
Friend support x walkability	0.048 (0.048) −0.05; 0.14 0.985	0.104 (0.044)* 0.02; 0.20 0.020	0.101 (0.046)* 0.01; 0.19 0.026	0.032 (0.055) −0.08; 0.14 0.559	−0.001 (0.014) −0.03; 0.03 0.994	−0.082 (0.029)* −0.14; −0.02 0.004
Social norm x walkability	−0.070 (0.070) −0.21; 0.07 0.319	0.070 (0.066) −0.06; 0.20 0.037	0.005 (0.067) −0.13; 0.14 0.941	−0.138 (0.078) ^¥^ −0.29; 0.02 0.078	−0.045 (0.022)* −0.09; −0.01 0.041	−0.056 (0.044) −0.14 0.03 0.195
Self-efficacy x walkability	−0.038 (0.052) −0.06; 0.14 0.467	0.129 (0.049)* 0.03; 0.23 0.008	0.019 (0.050) −0.08; 0.12 0.707	0.008 (0.056) −0.10; 0.12 0.883	0.001 (0.015) −0.03; 0.03 0.927	HI: −0.086 (0.051) ^¥^ −0.19; 0.01 0.092 LI: 0.151 (0.057)* 0.04; 0.26 0.008
Benefits x walkability	0.033 (0.074) −0.11; 0.18 0.651	0.029 (0.069) −0.11; 0.16 0.678	−0.020 (0.072) −0.16; 0.12 0.784	−0.065 (0.085) −0.23; 0.10 0.444	0.014 (0.024) −0.03; 0.06 0.558	HI: −0.096 (0.083) −0.26; 0.07 0.243 LI: 0.237 (0.082)* 0.08; 0.40 0.004
Barriers x walkability	0.041 (0.075) −0.11; 0.19 0.584	−0.068 (0.077) −0.22; 0.08 0.333	−0.091 (0.074) −0.24; 0.05 0.219	−0.047 (0.085) −0.21; 0.12 0.580	0.045 (0.024) ^¥^ −0.01; 0.09 0.063	0.028 (0.048) −0.07; 0.12 0.561
Psychosocial factors						
Attitude	LI: −0.056 (0.054) −0.16; 0.05 0.293 HI: 0.054 (0.056) −0.06; 0.17 0.343	0.091 (0.036)* 0.02; 0.16 0.012	0.056 (0.037) −0.02; 0.13 0.126	0.257 (0.043)** 0.18; 0.34 <0.001	0.042 (0.012)** 0.02; 0.07 <0.001	0.057 (0.025)* 0.01; 0.11 0.019
Parental support	−0.030 (0.035) −0.10; 0.04 0.382	0.073 (0.033)* 0.01; 0.14 0.026	0.087 (0.033)* 0.02; 0.15 0.007	0.360 (0.036)** 0.29; 0.43 <0.001	0.028 (0.011)* 0.01; 0.05 0.010	0.065 (0.021)* 0.02; 0.11 0.002
Friend support	−0.001 (0.024) −0.05; 0.05 0.953	0.037 (0.023) −0.01; 0.08 0.107	0.028 (0.023) −0.02; 0.07 0.231	0.060 (0.028)* 0.01; 0.12 0.031	0.023 (0.007)* 0.01; 0.04 0.001	0.035 (0.015)* 0.01; 0.06 0.019
Social norm	−0.067 (0.033)* −0.13; −0.01 0.044	0.056 (0.032) ^¥^ −0.01; 0.12 0.082	0.042 (0.032) −0.02; 0.10 0.184	0.207 (0.037)** 0.13; 0.28 <0.001	0.020 (0.011) ^¥^ −0.01; 0.04 0.061	0.028 (0.021) −0.01; 0.07 0.185
Self-efficacy	−0.018 (0.026) −0.07; 0.03 0.497	0.042 (0.025) ^¥^ ^−^0.01; 0.09 0.093	−0.003 (0.025) −0.05; 0.05 0.907	0.220 (0.028)** 0.16; 0.28 <0.001	0.042 (0.008)** 0.03; 0.06 <0.001	HI: 0.083 (0.021)** 0.04; 0.12 <0.001 LI: 0.026 (0.023) −0.02; 0.07 0.264
Benefits	−0.047 (0.037) −0.12; 0.03 0.205	0.127 (0.035)** 0.06: 0.20 <0.001	−0.004 (0.036) −0.07; 0.07 0.919	0.190 (0.043)** 0.11; 0.27 <0.001	0.037 (0.012)* 0.01; 0.06 0.003	HI: 0.004 (0.035) −0.06; 0.07 0.916 LI: 0.020 (0.035) −0.05; 0.09 0.556
Barriers	−0.004 (0.038) −0.08; 0.07 0.910	0.006 (0.037) −0.07; 0.08 0.860	0.002 (0.038) −0.07; 0.08 0.961	−0.269 (0.044)** −0.36; −0.18 <0.001	−0.038 (0.013)* −0.06; −0.02 0.002	−0.052 (0.025)* −0.10; −0.01 0.048

PA variables were logarithmically transformed

Analyses were controlled for sex, age, neighborhood SES, family SES and accelerometer wear time if relevant;

*CI* confidence intervals

LI: analyses conducted only in low-income neighborhoods because of significant interaction between walkability*neighborhood income*psychosocial factor

HI: analyses conducted in high-income neighborhoods because of significant interaction between walkability*neighborhood income*psychosocial factor

** *p* < 0.001; * *p* < 0.05; ¥ *p* < 0.10

**Table 4:** Multivariate associations between psychosocial factors, walkability and children’s PA

	Active transportation to school	Walking for transportation during leisure	Cycling for transportation during leisure	Sports during leisure	MVPA weekday	MVPA weekend day
	B(SE) 95% CI *p* LI *n* = 223 HI *n* = 232	B(SE) 95% CI*p* *n* = 406	B(SE) 95% CI *p* *n* = 457	B(SE) 95% CI *p* *n* = 409	B(SE) 95% CI*p* *n* = 375	B(SE) 95% CI *p* LI *n* = 152 HI *n* = 189
Psychosocial x walkability (ref = low)						
Parental attitude x walkability	LI: -0.351 (0.150)* −0.65; −0.06 0.019	−0.003 (0.080) −0.16; 0.15 0.971		0.051 (0.132) −0.21; 0.31 0.701	−0.072 (0.042) ^¥^ −0.15; 0.01 0.088	
Parental support x walkability				0.372 (0.060)* 0.25;0.49 0.018	−0.024 (0.026) −0.08; 0.03 0.355	
Friend support x walkability		0.055 (0.051) −0.05; 0.16 0.288	0.096 (0.046)* 0.01; 0.19 0.035			LI: −0.113 (0.055)* −0.22; −0.01 0.039 HI: −0.065 (0.062) −0.19; 0.06 0.296
Social norm x walkability				0.001 (0.088) −0.17; 0.17 0.987	−0.008 (0.027) −0.07; 0.05 0.769	
Self-efficacy x walkability		0.093 (0.054) ^¥^ −0.01; 0.20 0.088				LI:0.180 (0.071)* 0.04; 0.32 0.011 HI: −0.027 (0.065) −0.16; 0.10 0.682
Benefits x walkability						LI: 0.119 (0.090) −0.06; 0.30 0.186 HI: −0.085 (0.096) −0.27; 0.10 0.373
Barriers x walkability					0.014 (0.027) −0.04; 0.07 0.614	
Psychosocial factors						
Parental attitude		−0.003 (0.080) −0.16; 0.15 0.907		−0.053 (0.104) −0.26; 0.15 0.607	0.055 (0.031) ^¥^ −0.01; 0.12 0.071	LI:0.067 (0.057) −0.05; 0.18 0.242 HI: 0.038 (0.045) −0.05; 0.13 0.398
Parental support		0.049 (0.040) −0.03; 0.13 0.218	0.070 (0.033)* 0.01; 0.13 0.036	0.372 (0.060)** 0.25; 0.49 <0.001	0.014 (0.018) −0.02; 0.05 0.424	LI: −0.020 (0.033) −0.09; 0.05 0.557 HI: 0.066 (0.041) −0.01; 0.15 0.104
Friend support				−0.045 (0.028) −0.10; 0.01 0.105	0.014 (0.008) ^¥^ −0.01; 0.03 0.094	LI: 0.100 (0.049)* 0.01; 0.20 0.039 HI: 0.046 (0.026) ^¥^ −0.01; 0.10 0.078
Social norm	LI: −0.085 (0.048) ^¥^ −0.18; 0.01 0.079 HI: −0.036 (0.056) −0.15; 0.07 0.520	−0.007 (0.038) −0.08; 0.07 0.848		0.045 (0.072) −0.10; 0.19 0.534	0.001 (0.021) −0.04; 0.04 0.961	
Self-efficacy		−0.044 (0.039) −0.12;0.03 0.252		0.121 (0.034)** 0.05; 0.19 <0.001	0.033 (0.010)* 0.01; 0.05 0.001	HI: 0.055 (0.029) ^¥^ −0.01; 0.11 0.059
Benefits		0.091 (0.041)* 0.01; 0.17 0.026		0.028 (0.044) −0.06;0.11 0.527	0.010 (0.013) −0.02; 0.04 0.455	
Barriers				−0.145 (0.047)** −0.24; −0.05 <0.001	−0.018 (0.020) −0.06; 0.02 0.372	LI:0.032 (0.040) −0.05; 0.11 0.428 HI: −0.047 (0.046) −0.14; 0.04 0.310

This table included all independent variables with p < 0.10 in the bivariate analyses. All PA variables were logarithmically transformed

PA variables were logarithmically transformed

Analyses were controlled for sex, age, neighborhood SES, family SES and accelerometer wear time if relevant;

** *p* < 0.001; * *p* < 0.05; ^¥^ *p* < 0.10

LI: analyses conducted only in low-income neighborhoods because of significant interaction between walkability*neighborhood income*psychosocial factor

HI: analyses conducted in high-income neighborhoods because of significant interaction between walkability*neighborhood income*psychosocial factor

*CI* confidence interval

#### Active transportation to school

**Fig. 2:**
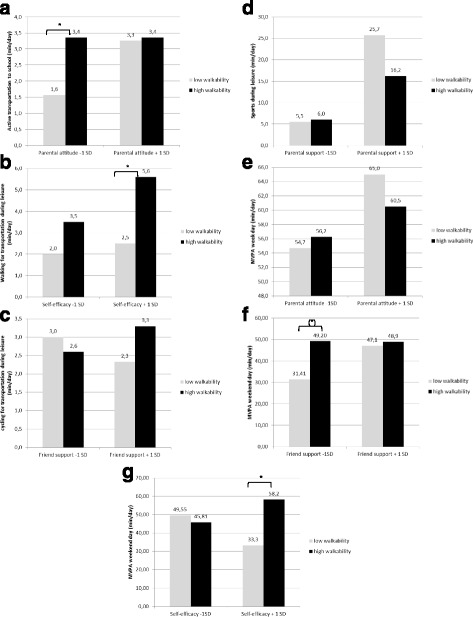
**a** Moderating effect of parental attitude in the association between walkability and active transportation to school. **b** Moderating effect of self-efficacy in the association between walkability and walking for transportation during leisure. **c** Moderating effect of friend support in the association between walkability and cycling for transportation. **d** Moderating effect of parental support in the association between walkability and sports during leisure. **e** Moderating effect of parental attitude in the association between walkability and MVPA on a weekday. **f** Moderating effect of friend support in the associatin between walkability and MVPA on a weekend day. **g** Moderating effect of self-efficacy in the assocation between walkability and MVPA on a weekend day

In low-income neighborhoods, parental attitude toward PA and walkability interacted in relation to active transportation to school (β = −0.351 ± 0.150, *p* = 0.019; Cohen’s f^2^ = 0.02; Fig. 2a). For children with low parental reported attitude, high vs. low walkability accounted for 1.80 additional min/day of walking for transportation during leisure. For children with high parental reported self-efficacy, high versus low walkability accounted for 0.10 additional min/day of walking for transportation during leisure.

In high income neighborhoods, no significant interaction effects and no significant associations of psychosocial factors and objective walkability with ATS were found (Table 4).

#### Walking for transportation during leisure

A moderating effect of parental reported self-efficacy was found in the relation between objective walkability and walking for transportation during leisure (β = 0.093 ± 0.054, *p* = 0.065; Cohen’s f^2^ < 0.001; Fig. 2b). For children with low parental reported self-efficacy, high vs. low walkability accounted for 1.50 additional min/day of walking for transportation during leisure. For children with high parental reported self-efficacy, high versus low walkability accounted for 3.10 additional min/day of walking for transportation during leisure.

Perceived benefits were positively related to walking for transportation during leisure (β = 0.091 ± 0.041, *p* = 0.026; Cohen’s f^2^ = 0.03) (Table 4).

#### Cycling for transportation during leisure

A moderating effect of friend support was found in the relation between walkability and cycling for transportation during leisure (β = 0.096 ± 0.046, *p* = 0.035; Cohen’s f^2^ < 0.001; Fig. 2c). For children with low friend support, high versus low walkability accounted for 0.40 fewer min/day of cycling for transportation during leisure. For children with high friend support, high versus low walkability accounted for 1.00 additional min/day of cycling for transportation during leisure.

Parental support was positively related to cycling for transportation during leisure (β = 0.070 ± 0.033, *p* = 0.036; Cohen’s f^2^ < 0.001) (Table 4).

#### Sports during leisure

A moderating effect of parental support was found in the relation between walkability and sports during leisure (β = 0.372 ± 0.060, *p* = 0.018; Cohen’s f^2^ = 0.01; Fig. 2d). For children with low parental support, high versus low walkability accounted for 0.50 additional min/day of sports during leisure. For children with high parental support, high versus low walkability accounted for 9.50 fewer min/day of sports during leisure.

Positive associations were found of parental reported parental support (β = 0.372 ± 0.060, *p* < 0.001; Cohen’s f^2^ = 0.10), and self-efficacy (β = 0.121 ± 0.034, *p* < 0.001; Cohen’s f^2^ = 0.04) with sports during leisure. A negative association was found between parental reported barriers and sports during leisure (β = -0.145 ± 0.047, *p* = 0.002; Cohen’s f^2^ = 0.02) (Table 4).

#### Objectively measured weekday MVPA

A moderating effect of parental attitude was found in the relation between walkability and MVPA on weekdays (β = −0.072 ± 0.042, *p* = 0.088; Cohen’s f^2^ < 0.001; Fig. 2e). For children with low parental attitude, high versus low walkability accounted for 1.5 additional min/day of MVPA on weekdays. For children with high parental support, high versus low walkability accounted for 4.50 fewer min/day of MVPA on weekdays.

Self-efficacy (β = 0.033 ± 0.010, *p* < 0.001; Cohen’s f^2^ < 0.001) was positively related to MVPA on weekdays (Table 4).

#### Objectively measured weekend day MVPA

In low-income neighborhoods, a moderating effect of parental reported friend support was found in the relation between walkability and MVPA on weekend days (β = −0.106 ± 0.054, *p* = 0.039; Cohen’s f^2^ = 0.04; Fig. 2f). For children with low friend support, high versus low walkability accounted for 17.79 additional minutes MVPA/weekend day. For children with high friend support, high versus low walkability accounted for 1.77 more minutes MVPA/weekend day. In addition, a moderating effect of parental reported self-efficacy was found in the relation between walkability and MVPA on weekend days (β = 0.180 ± 0.071, *p* = 0.011; Cohen’s f^2^ = 0.04; Fig. 2g) in low-income neighborhoods. For children with lower self-efficacy, high versus low walkability accounted for 3.74 fewer minutes MVPA/weekend day. For children with higher self-efficacy, high versus low walkability accounted for 24.29 more minutes MVPA/weekend day. Friend support was positively related to weekend MVPA (β = 0.100 ± 0.049, *p* = 0.04; Cohen’s f^2^ = 0.03).

In high-income neighborhoods, no significant interactions and main effects were found in the multivariate model (Table 4).

## Discussion

The main aim of this study was to investigate if psychosocial factors moderate the association between walkability and different domains of children’s PA. Furthermore, main effects of psychosocial factors on children’s PA were investigated.

Few interactions between neighborhood walkability and psychosocial factors were found in relation to different domains of PA. In total, only 7 out of 42 investigated interactions were significant and 3 of these interactions were only significant in low-income neighborhoods. Only 4 out of 42 interactions were significant in both high- and low-income neighborhoods. Besides, the effect sizes of these interactions were very small. This indicates that in general, there is no strong interaction between psychosocial factors and walkability in relation to children’s PA and in general, the hypothesis (i.e. that walkability would be more strongly related to PA among children with a negative psychosocial profile, whereas among children with a positive psychosocial profile walkability would be less important to explain PA) could not be not confirmed, as only few interactions in different directions were found. The lack of interactions between objective walkability and psychosocial factors in relation to children’s PA indicates that changing the objective walkability of a neighborhood might affect PA levels of children with positive versus negative psychosocial profiles in the same way. This was also found among Belgian older adults [26].

The hypothesis, based on a study among Belgian adolescents, that walkability would be more strongly related to PA among children with negative psychosocial profiles and that PA levels of children with a positive psychosocial profile would be high, irrespective of the neighborhood walkability, was only confirmed for 2 interactions among children living in low-income neighborhoods. Effect sizes of these interactions were small. Children living in low-income neighborhoods with lower parental attitude and lower friend support, engaged more in active transportation (+2 min/day) and in more MVPA on week days (+18 min/day) respectively, when they lived in a neighborhood with high walkability, compared to children living in low walkable neighborhoods. For children living in low-income neighborhoods, with higher friend support and a better parental attitude toward PA, walkability did not explain their PA. This shows that children who are at risk for lower PA levels, due to a lower SES [51] and a lower parental attitude and less friend support, would benefit the most of an increase in neighborhood walkability. However, these findings need to be confirmed in future research.

Also interactions in the opposite direction of the hypothesis (i.e. walkability was more strongly related to PA among children with positive psychosocial profiles compared to children with more negative psychosocial profiles) were found, but effect sizes of these interactions were mostly very small (Cohen’s f^2^ < 0.02). Only in one interaction that was found in the opposite direction of the hypothesis, a small effect size was found (Cohen’s f^2^ between 0.02 and 0.15). Among children with higher self-efficacy and living in low-income neighborhoods, a high walkable neighborhood accounted for 25 mins/day of MVPA per weekend day, whereas among children with a lower self-efficacy, walkability did not explain children’s weekend MVPA.

The low number of significant interactions between psychosocial factors and walkability in relation to PA and the small effect sizes of the significant interactions demonstrate that walkability interventions may affect children with different psychosocial profiles in the same way. So, based on previous findings, it might be presumed that increasing walkability will lead to more walking for transportation among children living in low-income neighborhoods. However, when walkability will be increased, it should be taken into account that this can also have negative effects on children’s sport during leisure in low-income neighborhoods [17]. Therefore, increasing walkability should focus on the increase of walkability for cyclists and pedestrians, but not for motorized traffic to retain the safety of the neighborhood. This is important as a safe neighborhood is related to more PA [7, 52, 53]. This can be done by making small streets only accessible for cyclists and pedestrians, but not for motorized traffic or by installing footbridges or underpasses for cyclists on busy and dangerous roads to increase the walkability. Although effect sizes in the current study were small, changing the neighborhood can affect large groups of children at the same time, which may result in beneficial effects.

A second aim of this study was to determine the main effects of psychosocial factors on children’s PA. Consistent main effects of psychosocial factors in relation to children’s PA with small effect sizes were found. It is possible that these small effect sizes are due to the fact that psychosocial factors were parental reported instead of children’s report. It is possible that larger effect sizes would have been found if children reported their psychosocial factors themselves. As expected, having a more positive psychosocial profile toward PA was related to more PA among children. The most important main effects were found in relation to children’s sports during leisure. Self-efficacy and parental support were directly positively related to children’s sports during leisure. Besides, although effect sizes were very small, parental support was also directly and positively related to cycling for transportation during leisure and self-efficacy was positively related to MVPA on weekdays. Furthermore, a direct and negative relation was found between perceived barriers and sports during leisure with a small effect size. This indicates that it might be valuable for parents living in high- and low-income neighborhoods and neighborhoods with high and low walkability to support their child to be physically active, by providing transportation to sports activities, by watching and cheering for their child during sports and by doing sports together. Furthermore, suggestions can be made to reduce barriers toward PA among children and to increase children’s self-efficacy, as perceiving many barriers and having low self-efficacy is related to less sports during leisure time. This can be done by offering children easy-accessible and enjoyable sport activities in terms of timing, location, costs and level such as extracurricular school-based sports [54]. Sport clubs should also focus on non-competitive sports in order to decrease the barriers ‘not liking sports’ and not ‘being good at sports’. Besides, children need to be made aware that they can be physically active, even if they have to get up early, if their friends want to do something else, if they have a lot of work for school and if PA is exhausting and difficult. Different behavior change techniques can be used to increase children’s self-efficacy (e.g. prompt barrier identification, action planning,..[55]). However, effect sizes of psychosocial factors in relation to children’s PA were small. Therefore, effects of changing these psychosocial factors are expected to be small, so changing psychosocial factors among children might not be the most ideal strategy to increase PA among children. In line with a review of previous studies [10], self-efficacy and parental support seem to be the two most important correlates of children’s sports and MVPA. Interventions focusing on the increase of parental support and self-efficacy and the decrease of barriers toward PA could possibly have small effects on children’s PA levels in high- and low-income neighborhoods and in neighborhoods with high or low walkability. Furthermore, future longitudinal research and intervention studies are necessary to confirm this hypothesis.

The relatively large sample, the combination of self-reported and objective assessment of children’s PA and the objective assessment of neighborhood walkability were strengths of this study. A first limitation of the study is the cross-sectional design as no causal relationships could be examined. By using cross-sectional data, it is possible to interpret the interactions in the other direction: i.e. walkability as a moderator of the association between psychosocial factors and PA. However, in the current manuscript interactions were described as if psychosocial factors moderate the association between walkability and PA, based on previous research among other age groups. Besides, a large number of associations between different factors and different domains of PA were investigated, due to the fact that hypotheses about the inclusion of variables could not be made due to lack of previous research and models. Therefore, before constructing the final model, a large number of preliminary bivariate analyses were conducted. Based on these preliminary analyses, the variables in the final model were determined. However, by investigating a large number of associations in the preliminary analyses, the likelihood of incorrectly rejecting a null hypothesis increased. The Bonferroni method is a method to counteract this problem, by lowering the significance level of a test (α = α/n, with n = the number of tests). However, an important disadvantage of using the Bonferroni correction is that this method is very strict and only focuses on reducing type 1 errors but the chance for making type 2 errors increases [56]. Furthermore, these analyses were executed as preliminary analyses, and the number of analyses and determinants in the final models were limited. Therefore, the Bonferroni correction was not applied to the analyses.

Furthermore, in Ghent, more people live in high walkable, low-income neighborhoods (*n* = 157389) or low walkable, high income neighborhoods (*n* = 44809) compared to low walkable, low-income neighborhoods (*n* = 9623) or high walkable, high income neighborhoods (*n* = 43733). As children were recruited in schools instead of neighborhoods with varying walkability and income levels, this led to the inclusion of children living mostly in high-income, low walkable or low-income, high walkable neighborhoods; children living in low-income, low walkable and high-income, high walkable neighborhoods were underrepresented. So the division of children across the different neighborhoods in the current study represents the actual distribution of children living in these neighborhoods but this is a methodological weakness of the study. Besides, response rates of school principals were rather low, however this was comparable to prior studies [57] that were based on questionnaires for pupils and parents. The low response rate might be due to the fact that schools have many obligations and are consequently not very keen on spending time on research activities. It is possible that parents who attach more importance to PA could have been more willing to let their child participate in this study*.* Furthermore, psychosocial factors were formulated toward PA and sports in general and not toward specific domains of PA (e.g. walking for transportation during leisure). It is likely that the formulation of these factors toward active transportation would have resulted in more significant associations between these factors and domains of active transportation. Therefore, it is recommended for future research to formulate the psychosocial factors more specifically toward the domain of PA that is investigated. Psychosocial factors were also mainly questioned from the parents’ viewpoint rather than from children’s viewpoint. Parental report of psychosocial factors was used in previous studies with acceptable reliability [37, 38]. Furthermore, parental education was used as a proxy measure of family SES, as data on working or income status were unavailable in this study. Future research is necessary as it is likely that other factors also moderate the association between objective walkability and PA.

## Conclusions

Only few interactions (7 out of 42), with very small effect sizes between objective neighborhood walkability and psychosocial characteristics were found in relation to children’s PA in different directions. The hypothesis that walkability would be more strongly related to PA among children with more negative psychosocial profiles could not be confirmed. Increasing walkability for cyclists and pedestrians might be effective in increasing PA among children with positive and negative psychosocial profiles toward PA. In both high- and low-income neighborhoods, parental support and self-efficacy were positively related to children’s sports and MVPA, independent of the level of walkability. Based on the results of this study, it seems that focusing on these specific psychosocial factors to increase PA can possibly be effective in both high- and low-walkable neighborhoods, as well as in high- and low-income neighborhoods, although the effects can be small.

## Additional file

Additional file 1: Supplementary material. (SAV 105 kb)

## Abbreviations

ATS: Active transportation to school
BEPAS: Belgian Environmental Physical Activity Study
FPAQ: Flemish Physical Activity Questionnaire
GIS: Geographical information system
MVPA: Moderate- to vigorous-intensity physical activity
PA: Physical activity
SES: Socio-economic status
